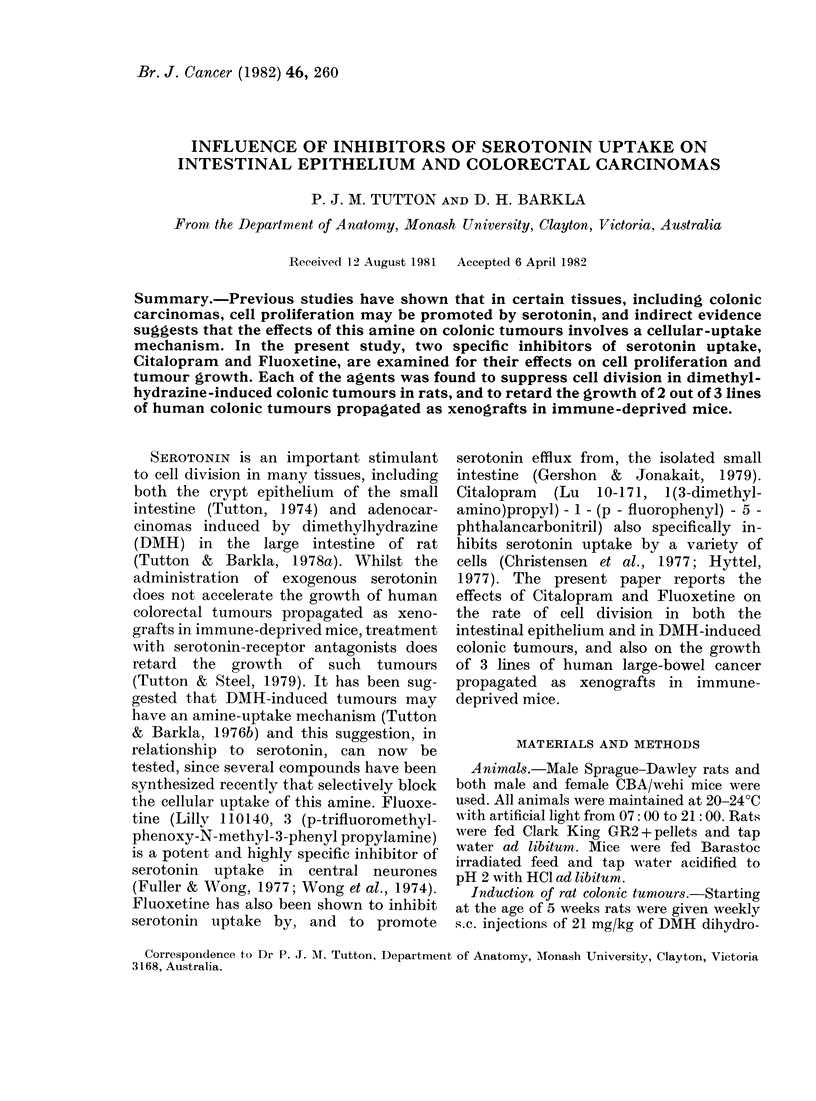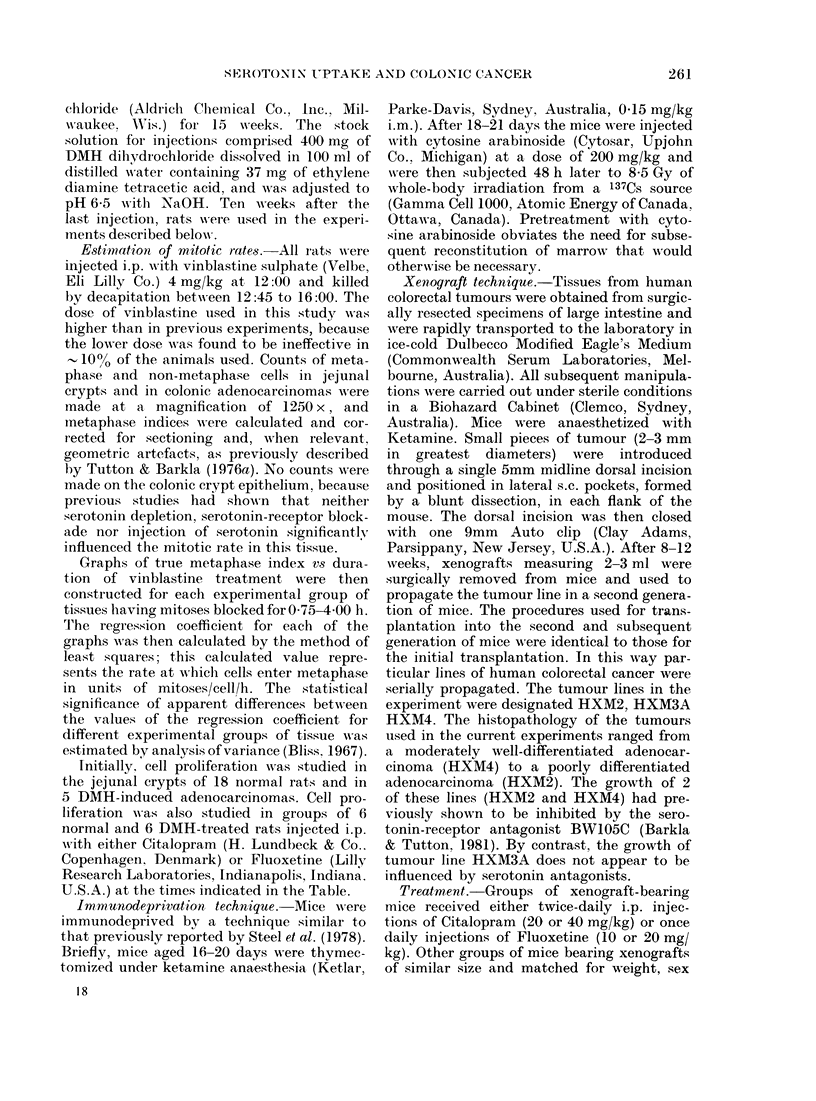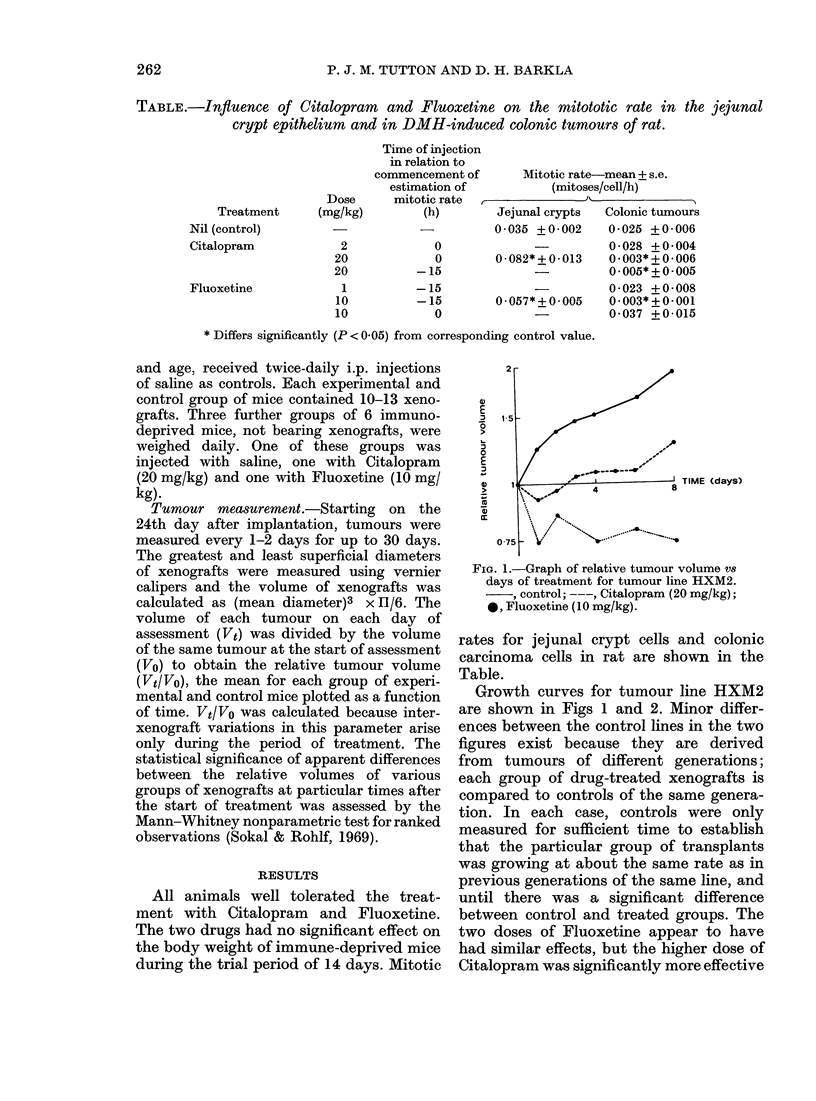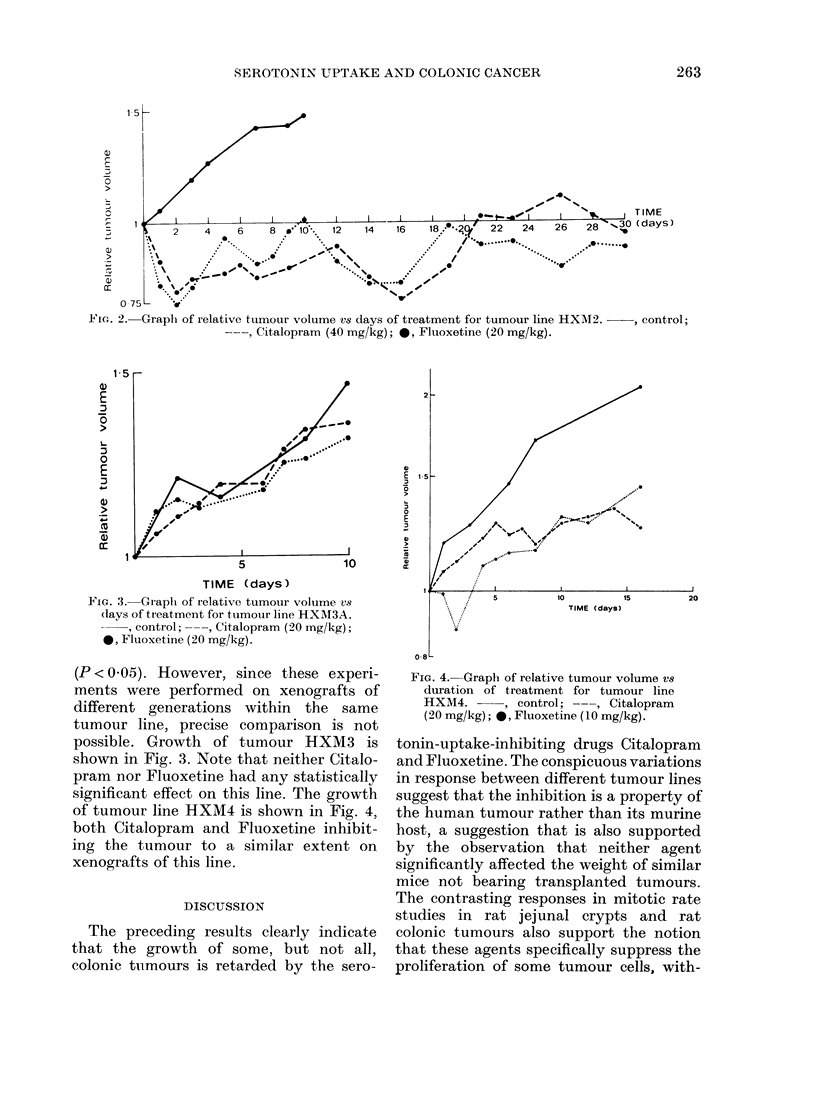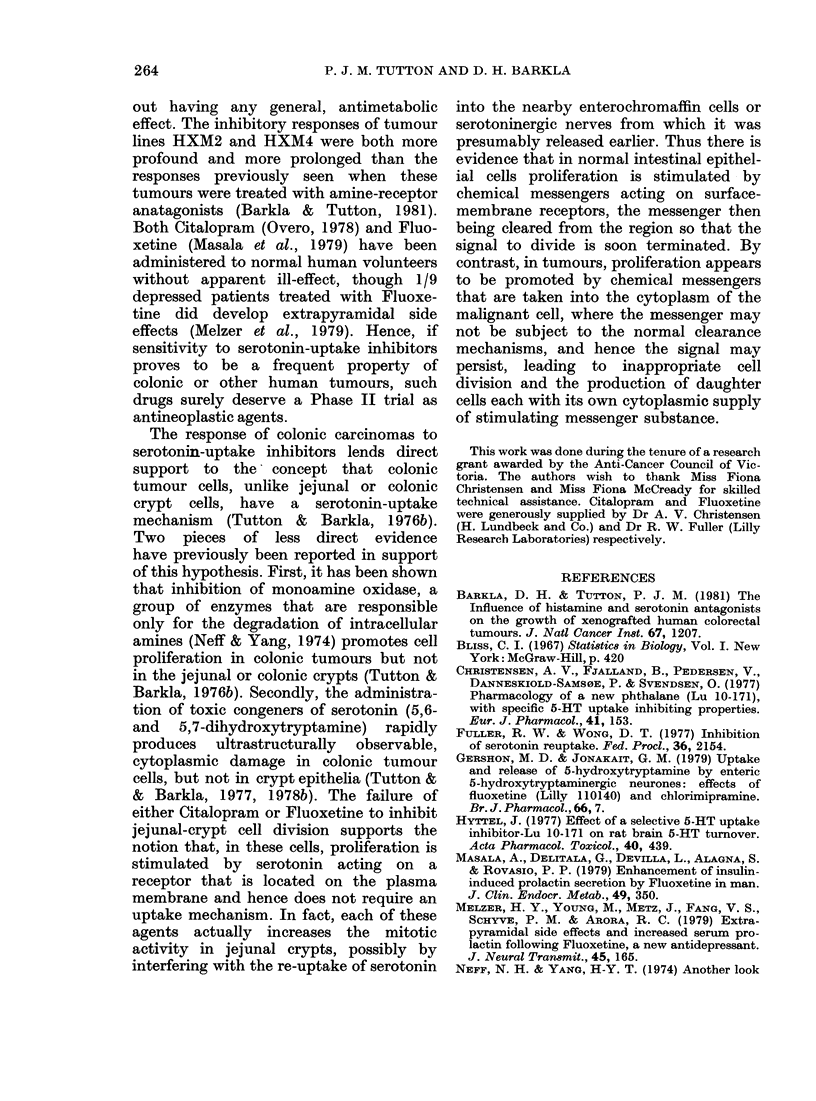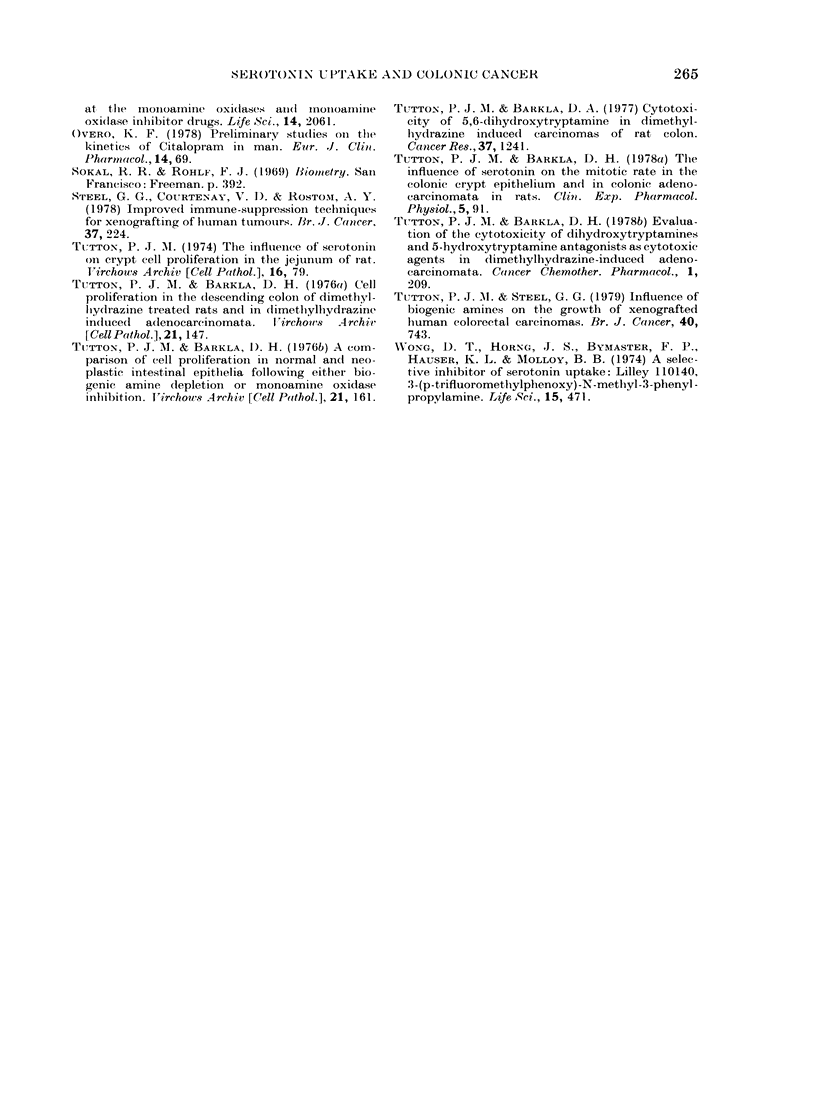# Influence of inhibitors of serotonin uptake on intestinal epithelium and colorectal carcinomas.

**DOI:** 10.1038/bjc.1982.191

**Published:** 1982-08

**Authors:** P. J. Tutton, D. H. Barkla

## Abstract

Previous studies have shown that in certain tissues, including colonic carcinomas, cell proliferation may be promoted by serotonin, and indirect evidence suggests that the effects of this amine on colonic tumours involves a cellular-uptake mechanism. In the present study, two specific inhibitors of serotonin uptake, Citalopram and Fluoxetine, are examined for their effects on cell proliferation and tumour growth. Each of the agents was found to suppress cell division in dimethylhydrazine-induced colonic tumours in rats, and to retard the growth of 2 out of 3 lines of human colonic tumours propagated as xenografts in immune-deprived mice.


*Br. J. Cancer* (1982) **46**, 260

### INFLUENCE OF INHIBITORS OF SEROTONIN UPTAKE ON INTESTINAL EPITHELIUM AND COLORECTAL CARCINOMAS

P. J. M. TUTTON AND D. H. BARKLA

*From the Department of Anatomy, Monash University, Clayton, Victoria, Australia*

Received 12 August 1981 Accepted 6 April 1982

**Summary.—Previous studies have shown that in certain tissues, including colonic carcinomas, cell proliferation may be promoted by serotonin, and indirect evidence suggests that the effects of this amine on colonic tumours involves a cellular-uptake mechanism. In the present study, two specific inhibitors of serotonin uptake, Citalopram and Fluoxetine, are examined for their effects on cell proliferation and tumour growth. Each of the agents was found to suppress cell division in dimethylhydrazine-induced colonic tumours in rats, and to retard the growth of 2 out of 3 lines of human colonic tumours propagated as xenografts in immune-deprived mice.**

SEROTONIN is an important stimulant to cell division in many tissues, including both the crypt epithelium of the small intestine (Tutton, 1974) and adenocarcinomas induced by dimethylhydrazine (DMH) in the large intestine of rat (Tutton & Barkla, 1978*a*). Whilst the administration of exogenous serotonin does not accelerate the growth of human colorectal tumours propagated as xenografts in immune-deprived mice, treatment with serotonin-receptor antagonists does retard the growth of such tumours (Tutton & Steel, 1979). It has been suggested that DMH-induced tumours may have an amine-uptake mechanism (Tutton & Barkla, 1976*b*) and this suggestion, in relationship to serotonin, can now be tested, since several compounds have been synthesized recently that selectively block the cellular uptake of this amine. Fluoxetine (Lilly 110140, 3 (p-trifluoromethylphenoxy-N-methyl-3-phenyl propylamine) is a potent and highly specific inhibitor of serotonin uptake in central neurones (Fuller & Wong, 1977; Wong *et al.*, 1974). Fluoxetine has also been shown to inhibit serotonin uptake by, and to promote serotonin efflux from, the isolated small intestine (Gershon & Jonakait, 1979). Citalopram (Lu 10-171, 1(3-dimethylamino)propyl) - 1 - (p - fluorophenyl) - 5 - phthalancarbonitril) also specifically inhibits serotonin uptake by a variety of cells (Christensen *et al.*, 1977; Hyttel, 1977). The present paper reports the effects of Citalopram and Fluoxetine on the rate of cell division in both the intestinal epithelium and in DMH-induced colonic tumours, and also on the growth of 3 lines of human large-bowel cancer propagated as xenografts in immune-deprived mice.

### MATERIALS AND METHODS

*Animals*.—Male Sprague–Dawley rats and both male and female CBA/wehi mice were used. All animals were maintained at 20–24°C with artificial light from 07 : 00 to 21 : 00. Rats were fed Clark King GR2+pellets and tap water *ad libitum*. Mice were fed Barastoc irradiated feed and tap water acidified to pH 2 with HCl *ad libitum*.

*Induction of rat colonic tumours*.—Starting at the age of 5 weeks rats were given weekly s.c. injections of 21 mg/kg of DMH dihydro-

Correspondence to Dr P. J. M. Tutton, Department of Anatomy, Monash University, Clayton, Victoria 3168, Australia.

chloride (Aldrich Chemical Co., Inc., Milwaukee, Wis.) for 15 weeks. The stock solution for injections comprised 400 mg of DMH dihydrochloride dissolved in 100 ml of distilled water containing 37 mg of ethylene diamine tetracetic acid, and was adjusted to pH 6·5 with NaOH. Ten weeks after the last injection, rats were used in the experiments described below.

*Estimation of mitotic rates.*—All rats were injected i.p. with vinblastine sulphate (Velbe, Eli Lilly Co.) 4 mg/kg at 12:00 and killed by decapitation between 12:45 to 16:00. The dose of vinblastine used in this study was higher than in previous experiments, because the lower dose was found to be ineffective in ~10% of the animals used. Counts of metaphase and non-metaphase cells in jejunal crypts and in colonic adenocarcinomas were made at a magnification of 1250×, and metaphase indices were calculated and corrected for sectioning and, when relevant, geometric artefacts, as previously described by Tutton & Barkla (1976*a*). No counts were made on the colonic crypt epithelium, because previous studies had shown that neither serotonin depletion, serotonin-receptor blockade nor injection of serotonin significantly influenced the mitotic rate in this tissue.

Graphs of true metaphase index *vs* duration of vinblastine treatment were then constructed for each experimental group of tissues having mitoses blocked for 0·75–4·00 h. The regression coefficient for each of the graphs was then calculated by the method of least squares; this calculated value represents the rate at which cells enter metaphase in units of mitoses/cell/h. The statistical significance of apparent differences between the values of the regression coefficient for different experimental groups of tissue was estimated by analysis of variance (Bliss, 1967).

Initially, cell proliferation was studied in the jejunal crypts of 18 normal rats and in 5 DMH-induced adenocarcinomas. Cell proliferation was also studied in groups of 6 normal and 6 DMH-treated rats injected i.p. with either Citalopram (H. Lundbeck & Co., Copenhagen, Denmark) or Fluoxetine (Lilly Research Laboratories, Indianapolis, Indiana, U.S.A.) at the times indicated in the Table.

*Immunodeprivation technique.*—Mice were immunodeprived by a technique similar to that previously reported by Steel *et al.* (1978). Briefly, mice aged 16–20 days were thymectomized under ketamine anaesthesia (Ketlar, Parke-Davis, Sydney, Australia, 0·15 mg/kg i.m.). After 18–21 days the mice were injected with cytosine arabinoside (Cytosar, Upjohn Co., Michigan) at a dose of 200 mg/kg and were then subjected 48 h later to 8·5 Gy of whole-body irradiation from a $^{137}Cs$ source (Gamma Cell 1000, Atomic Energy of Canada, Ottawa, Canada). Pretreatment with cytosine arabinoside obviates the need for subsequent reconstitution of marrow that would otherwise be necessary.

*Xenograft technique.*—Tissues from human colorectal tumours were obtained from surgically resected specimens of large intestine and were rapidly transported to the laboratory in ice-cold Dulbecco Modified Eagle's Medium (Commonwealth Serum Laboratories, Melbourne, Australia). All subsequent manipulations were carried out under sterile conditions in a Biohazard Cabinet (Clemco, Sydney, Australia). Mice were anaesthetized with Ketamine. Small pieces of tumour (2–3 mm in greatest diameters) were introduced through a single 5mm midline dorsal incision and positioned in lateral s.c. pockets, formed by a blunt dissection, in each flank of the mouse. The dorsal incision was then closed with one 9mm Auto clip (Clay Adams, Parsippany, New Jersey, U.S.A.). After 8–12 weeks, xenografts measuring 2–3 ml were surgically removed from mice and used to propagate the tumour line in a second generation of mice. The procedures used for transplantation into the second and subsequent generation of mice were identical to those for the initial transplantation. In this way particular lines of human colorectal cancer were serially propagated. The tumour lines in the experiment were designated HXM2, HXM3A HXM4. The histopathology of the tumours used in the current experiments ranged from a moderately well-differentiated adenocarcinoma (HXM4) to a poorly differentiated adenocarcinoma (HXM2). The growth of 2 of these lines (HXM2 and HXM4) had previously shown to be inhibited by the serotonin-receptor antagonist BW105C (Barkla & Tutton, 1981). By contrast, the growth of tumour line HXM3A does not appear to be influenced by serotonin antagonists.

*Treatment.*—Groups of xenograft-bearing mice received either twice-daily i.p. injections of Citalopram (20 or 40 mg/kg) or once daily injections of Fluoxetine (10 or 20 mg/kg). Other groups of mice bearing xenografts of similar size and matched for weight, sex

TABLE.—*Influence of Citalopram and Fluoxetine on the mitototic rate in the jejunal crypt epithelium and in DMH-induced colonic tumours of rat.*

| Treatment | Dose (mg/kg) | Time of injection in relation to commencement of estimation of mitotic rate (h) | Mitotic rate—mean ± s.e. (mitoses/cell/h) Jejunal crypts | Colonic tumours |
|---|---|---|---|---|
| Nil (control) | — | — | 0·035 ±0·002 | 0·025 ±0·006 |
| Citalopram | 2 | 0 | — | 0·028 ±0·004 |
| | 20 | 0 | 0·082* ±0·013 | 0·003* ±0·006 |
| | 20 | −15 | — | 0·005* ±0·005 |
| Fluoxetine | 1 | −15 | — | 0·023 ±0·008 |
| | 10 | −15 | 0·057* ±0·005 | 0·003* ±0·001 |
| | 10 | 0 | — | 0·037 ±0·015 |

* Differs significantly ($P < 0{\cdot}05$) from corresponding control value.

and age, received twice-daily i.p. injections of saline as controls. Each experimental and control group of mice contained 10–13 xenografts. Three further groups of 6 immunodeprived mice, not bearing xenografts, were weighed daily. One of these groups was injected with saline, one with Citalopram (20 mg/kg) and one with Fluoxetine (10 mg/kg).

*Tumour measurement.*—Starting on the 24th day after implantation, tumours were measured every 1–2 days for up to 30 days. The greatest and least superficial diameters of xenografts were measured using vernier calipers and the volume of xenografts was calculated as (mean diameter)$^3$ $\times \Pi/6$. The volume of each tumour on each day of assessment ($V_t$) was divided by the volume of the same tumour at the start of assessment ($V_0$) to obtain the relative tumour volume ($V_t/V_0$), the mean for each group of experimental and control mice plotted as a function of time. $V_t/V_0$ was calculated because inter-xenograft variations in this parameter arise only during the period of treatment. The statistical significance of apparent differences between the relative volumes of various groups of xenografts at particular times after the start of treatment was assessed by the Mann–Whitney nonparametric test for ranked observations (Sokal & Rohlf, 1969).

RESULTS

All animals well tolerated the treatment with Citalopram and Fluoxetine. The two drugs had no significant effect on the body weight of immune-deprived mice during the trial period of 14 days. Mitotic

FIG. 1.—Graph of relative tumour volume *vs* days of treatment for tumour line HXM2. ——, control; ----, Citalopram (20 mg/kg); ●, Fluoxetine (10 mg/kg).

rates for jejunal crypt cells and colonic carcinoma cells in rat are shown in the Table.

Growth curves for tumour line HXM2 are shown in Figs 1 and 2. Minor differences between the control lines in the two figures exist because they are derived from tumours of different generations; each group of drug-treated xenografts is compared to controls of the same generation. In each case, controls were only measured for sufficient time to establish that the particular group of transplants was growing at about the same rate as in previous generations of the same line, and until there was a significant difference between control and treated groups. The two doses of Fluoxetine appear to have had similar effects, but the higher dose of Citalopram was significantly more effective

FIG. 2.—Graph of relative tumour volume *vs* days of treatment for tumour line HXM2. ——, control; – – –, Citalopram (40 mg/kg); ●, Fluoxetine (20 mg/kg).

FIG. 3.—Graph of relative tumour volume *vs* days of treatment for tumour line HXM3A. ——, control; – – –, Citalopram (20 mg/kg); ●, Fluoxetine (20 mg/kg).

FIG. 4.—Graph of relative tumour volume *vs* duration of treatment for tumour line HXM4. ——, control; – – –, Citalopram (20 mg/kg); ●, Fluoxetine (10 mg/kg).

($P < 0{\cdot}05$). However, since these experiments were performed on xenografts of different generations within the same tumour line, precise comparison is not possible. Growth of tumour HXM3 is shown in Fig. 3. Note that neither Citalopram nor Fluoxetine had any statistically significant effect on this line. The growth of tumour line HXM4 is shown in Fig. 4, both Citalopram and Fluoxetine inhibiting the tumour to a similar extent on xenografts of this line.

### DISCUSSION

The preceding results clearly indicate that the growth of some, but not all, colonic tumours is retarded by the serotonin-uptake-inhibiting drugs Citalopram and Fluoxetine. The conspicuous variations in response between different tumour lines suggest that the inhibition is a property of the human tumour rather than its murine host, a suggestion that is also supported by the observation that neither agent significantly affected the weight of similar mice not bearing transplanted tumours. The contrasting responses in mitotic rate studies in rat jejunal crypts and rat colonic tumours also support the notion that these agents specifically suppress the proliferation of some tumour cells, without having any general, antimetabolic effect. The inhibitory responses of tumour lines HXM2 and HXM4 were both more profound and more prolonged than the responses previously seen when these tumours were treated with amine-receptor anatagonists (Barkla & Tutton, 1981). Both Citalopram (Overo, 1978) and Fluoxetine (Masala *et al.*, 1979) have been administered to normal human volunteers without apparent ill-effect, though 1/9 depressed patients treated with Fluoxetine did develop extrapyramidal side effects (Melzer *et al.*, 1979). Hence, if sensitivity to serotonin-uptake inhibitors proves to be a frequent property of colonic or other human tumours, such drugs surely deserve a Phase II trial as antineoplastic agents.

The response of colonic carcinomas to serotonin-uptake inhibitors lends direct support to the concept that colonic tumour cells, unlike jejunal or colonic crypt cells, have a serotonin-uptake mechanism (Tutton & Barkla, 1976*b*). Two pieces of less direct evidence have previously been reported in support of this hypothesis. First, it has been shown that inhibition of monoamine oxidase, a group of enzymes that are responsible only for the degradation of intracellular amines (Neff & Yang, 1974) promotes cell proliferation in colonic tumours but not in the jejunal or colonic crypts (Tutton & Barkla, 1976*b*). Secondly, the administration of toxic congeners of serotonin (5,6- and 5,7-dihydroxytryptamine) rapidly produces ultrastructurally observable, cytoplasmic damage in colonic tumour cells, but not in crypt epithelia (Tutton & & Barkla, 1977, 1978*b*). The failure of either Citalopram or Fluoxetine to inhibit jejunal-crypt cell division supports the notion that, in these cells, proliferation is stimulated by serotonin acting on a receptor that is located on the plasma membrane and hence does not require an uptake mechanism. In fact, each of these agents actually increases the mitotic activity in jejunal crypts, possibly by interfering with the re-uptake of serotonin into the nearby enterochromaffin cells or serotoninergic nerves from which it was presumably released earlier. Thus there is evidence that in normal intestinal epithelial cells proliferation is stimulated by chemical messengers acting on surface-membrane receptors, the messenger then being cleared from the region so that the signal to divide is soon terminated. By contrast, in tumours, proliferation appears to be promoted by chemical messengers that are taken into the cytoplasm of the malignant cell, where the messenger may not be subject to the normal clearance mechanisms, and hence the signal may persist, leading to inappropriate cell division and the production of daughter cells each with its own cytoplasmic supply of stimulating messenger substance.

This work was done during the tenure of a research grant awarded by the Anti-Cancer Council of Victoria. The authors wish to thank Miss Fiona Christensen and Miss Fiona McCready for skilled technical assistance. Citalopram and Fluoxetine were generously supplied by Dr A. V. Christensen (H. Lundbeck and Co.) and Dr R. W. Fuller (Lilly Research Laboratories) respectively.

### REFERENCES

BARKLA, D. H. & TUTTON, P. J. M. (1981) The Influence of histamine and serotonin antagonists on the growth of xenografted human colorectal tumours. *J. Natl Cancer Inst.* **67**, 1207.

BLISS, C. I. (1967) *Statistics in Biology*, Vol. I. New York: McGraw-Hill, p. 420

CHRISTENSEN, A. V., FJALLAND, B., PEDERSEN, V., DANNESKIOLD-SAMSØE, P. & SVENDSEN, O. (1977) Pharmacology of a new phthalane (Lu 10-171), with specific 5-HT uptake inhibiting properties. *Eur. J. Pharmacol.*, **41**, 153.

FULLER, R. W. & WONG, D. T. (1977) Inhibition of serotonin reuptake. *Fed. Procl.*, **36**, 2154.

GERSHON, M. D. & JONAKAIT, G. M. (1979) Uptake and release of 5-hydroxytryptamine by enteric 5-hydroxytryptaminergic neurones: effects of fluoxetine (Lilly 110140) and chlorimipramine. *Br. J. Pharmacol.*, **66**, 7.

HYTTEL, J. (1977) Effect of a selective 5-HT uptake inhibitor-Lu 10-171 on rat brain 5-HT turnover. *Acta Pharmacol. Toxicol.*, **40**, 439.

MASALA, A., DELITALA, G., DEVILLA, L., ALAGNA, S. & ROVASIO, P. P. (1979) Enhancement of insulin-induced prolactin secretion by Fluoxetine in man. *J. Clin. Endocr. Metab.*, **49**, 350.

MELZER, H. Y., YOUNG, M., METZ, J., FANG, V. S., SCHYVE, P. M. & ARORA, R. C. (1979) Extrapyramidal side effects and increased serum prolactin following Fluoxetine, a new antidepressant. *J. Neural Transmit.*, **45**, 165.

NEFF, N. H. & YANG, H-Y. T. (1974) Another look at the monoamine oxidases and monoamine oxidase inhibitor drugs. *Life Sci.*, **14**, 2061.

OVERO, K. F. (1978) Preliminary studies on the kinetics of Citalopram in man. *Eur. J. Clin. Pharmacol.*, **14**, 69.

SOKAL, R. R. & ROHLF, F. J. (1969) *Biometry*. San Francisco: Freeman. p. 392.

STEEL, G. G., COURTENAY, V. D. & ROSTOM, A. Y. (1978) Improved immune-suppression techniques for xenografting of human tumours. *Br. J. Cancer*, **37**, 224.

TUTTON, P. J. M. (1974) The influence of serotonin on crypt cell proliferation in the jejunum of rat. *Virchows Archiv* [*Cell Pathol.*], **16**, 79.

TUTTON, P. J. M. & BARKLA, D. H. (1976*a*) Cell proliferation in the descending colon of dimethylhydrazine treated rats and in dimethylhydrazine induced adenocarcinomata. *Virchows Archiv* [*Cell Pathol.*], **21**, 147.

TUTTON, P. J. M. & BARKLA, D. H. (1976*b*) A comparison of cell proliferation in normal and neoplastic intestinal epithelia following either biogenic amine depletion or monoamine oxidase inhibition. *Virchows Archiv* [*Cell Pathol.*], **21**, 161.

TUTTON, P. J. M. & BARKLA, D. A. (1977) Cytotoxicity of 5,6-dihydroxytryptamine in dimethylhydrazine induced carcinomas of rat colon. *Cancer Res.*, **37**, 1241.

TUTTON, P. J. M. & BARKLA, D. H. (1978*a*) The influence of serotonin on the mitotic rate in the colonic crypt epithelium and in colonic adenocarcinomata in rats. *Clin. Exp. Pharmacol. Physiol.*, **5**, 91.

TUTTON, P. J. M. & BARKLA, D. H. (1978*b*) Evaluation of the cytotoxicity of dihydroxytryptamines and 5-hydroxytryptamine antagonists as cytotoxic agents in dimethylhydrazine-induced adenocarcinomata. *Cancer Chemother. Pharmacol.*, **1**, 209.

TUTTON, P. J. M. & STEEL, G. G. (1979) Influence of biogenic amines on the growth of xenografted human colorectal carcinomas. *Br. J. Cancer*, **40**, 743.

WONG, D. T., HORNG, J. S., BYMASTER, F. P., HAUSER, K. L. & MOLLOY, B. B. (1974) A selective inhibitor of serotonin uptake: Lilley 110140, 3-(p-trifluoromethylphenoxy)-N-methyl-3-phenylpropylamine. *Life Sci.*, **15**, 471.